# Associations of Metabolically Healthy Obesity with Gastroesophageal Reflux Disease and Ineffective Esophageal Motility

**DOI:** 10.5152/tjg.2025.24351

**Published:** 2025-02-11

**Authors:** Tao He, Li-Ping Su, Shun-Zhe Song, Yu-Fei Li, Li-Xia Wang, Shan-Ming Sun

**Affiliations:** 1 Department of Gastroenterology, The First Affiliated Hospital of Dalian Medical University, Liaoning, China; 2 Department of Gastroenterology, Weifang People’s Hospital, Shandong Second Medical University, Shandong, China

**Keywords:** Ambulatory 24-hour esophageal pH monitoring, high-resolution manometry, gastroesophageal reflux disease, ineffective esophageal motility, metabolically healthy obesity

## Abstract

**Background/Aims:**

Obesity correlates with a higher prevalence of gastroesophageal reflux disease (GERD) and ineffective esophageal motility (IEM); however, the connection between metabolic obesity phenotype and these symptoms is poorly explored. Here, empirical data were used to explore the relationships between phenotypes of metabolic obesity and GERD and IEM.

**Materials and Methods:**

The present retrospective study involved 605 patients demonstrating typical reflux symptoms, categorized into 4 phenotypes: metabolically healthy obesity (MHO), metabolically healthy non-obesity (MHNO), metabolically unhealthy obesity (MUO), and metabolically unhealthy non-obesity (MUNO). The study excluded cases who were underweight, with severe comorbidities, prior gastric surgeries, or an absence of complete data. A 24-hour multichannel intraluminal impedance-pH system was used for monitoring.

**Results:**

Patients exhibiting MUO, MHO, and MUNO phenotypes demonstrated a higher risk of GERD (pathological acid exposure time (AET), >6%) and IEM compared to those with the MHNO phenotype. Potential confounders, such as sex, age, body mass index, waist–hip ratio, smoking status, alcohol intake, psychosocial stress, socioeconomic status, dietary practices, and opioid usage were adjusted, with the results indicating that the MUO phenotype was linked to the highest risk of pathological AET [15.78 (95% CI: 4.72-52.73)]; IEM [3.00 (95% CI: 1.31-6.87)].

**Conclusion:**

The effects of obesity on GERD and IEM incidence could exceed those of metabolic diseases.

**Main Points**

- Obesity correlates with increased gastroesophageal reflux disease (GERD) and ineffective esophageal motility (IEM) prevalence; however, the connection between metabolic obesity phenotype and these symptoms is not well understood.
- Individuals with a metabolically healthy obesity phenotype were more likely to experience GERD and IEM than those who were metabolically healthy and not obese.
- It is suggested that all obese people, regardless of their metabolic status, should manage their weight.

## Introduction

Gastroesophageal reflux disease (GERD) involves the esophageal regurgitation of stomach acid, leading to heartburn. This results in complications, including Barrett’s esophagus (BE), reflux esophagitis (RE), and peptic stricture.^1^ The GERD incidence has risen recently due to alterations in diet and lifestyle. Furthermore, GERD negatively affects health-related quality of life, and its widespread incidence, coupled with its chronic nature, leads to significant healthcare expenditures. Understanding the underlying mechanisms of GERD is essential for implementing preventative measures.

Obesity, a significant worldwide public health concern, has a major influence on GERD development. Previous research has demonstrated that the prevalence of GERD is correlated with a raised body mass index (BMI).^2^ Furthermore, Rogers et al^3^ discovered that the probability of suffering from esophageal reflux symptoms and abnormal reflux durations escalates with increasing BMI. Additionally, esophageal motility disorders other than GERD have been observed in a high proportion of obese patients.^4^ A higher abnormal esophageal function, high-resolution manometry (HRM), abnormal esophageal contractility, gastroesophageal pressure gradient, and abnormal esophagogastric junction (EGJ) morphology results that suggest a higher risk of reflux were also present in these conditions.^4^ An additional investigation indicated that the association between BMI and reflux is mainly attributable to pressure.^5^ Several metabolic disorders, such as hyperglycemia,^6^ hypertension, and dyslipidemia, are linked to obesity, while metabolic syndrome has been linked to GERD.^7^ Obese individuals show distinct metabolic features, and the ramifications of these changes for GERD are little understood. The metabolically healthy obesity (MHO) phenotype is seen in obese people lacking metabolic abnormalities.^8^

Earlier research has shown the correlation between obesity and GERD, yet there is a lack of data regarding esophageal reflux and motility among patients carrying an MHO phenotype. The current investigation evaluated esophageal function in people with different metabolic obesity phenotypes using various techniques, including endoscopy and monitoring with a 24-hour multichannel intraluminal impedance-pH (24-h MII-pH) device. The study sought to offer perspectives for developing clinical prevention and intervention strategies customized to patients with varying metabolic obesity phenotypes.

## Materials and Methods

### Participants

**Figure 1.**
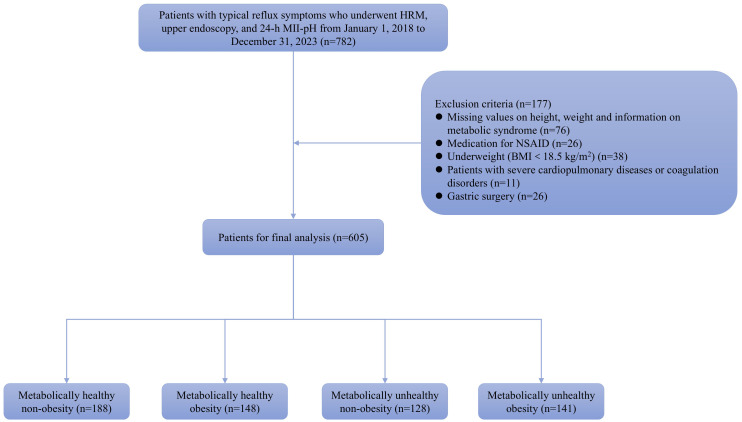
Flowchart demonstrating the study design.

This retrospective study included 782 consecutive patients treated at the Gastrointestinal Motility Center between January 1, 2018, and December 31, 2023, who had heartburn or regurgitation for a minimum of 3 months. Every patient had HRM, upper endoscopy, and 24-h MII-pH monitoring. The requirements for exclusion consisted of the following: earlier experience of gastric operation (n = 26), medication with non-steroidal anti-inflammatory drugs (n = 26), coagulation disorders or severe cardiopulmonary diseases (n = 11), lacking metabolic syndrome data, height, or weight (n = 76), and underweight status (n = 38, BMI < 18.5 kg/m^2^). Eventually, 605 participants were recruited into the research and categorized into 4 groups based on phenotype: metabolically unhealthy obesity (MUO), MHO, metabolically healthy non-obesity (MHNO), and metabolically unhealthy non-obesity (MUNO) (Figure 1). The present research complied with the Declaration of Helsinki and received approval (Approval Number: PJ-KS-KY-2023-33; Date: January 9, 2023) from the Medical Science Research Ethics Committee of the First Affiliated Hospital of Dalian Medical University. Every patient provided written informed consent.

### Anthropometric Measurements

At the initial presentation, the patient’s consumption of drugs, medical history, biochemical data, and demographics were documented. Demographic characteristics included smoking, hip circumference, height, body weight, sex, age, waist circumference, alcohol consumption, physical activity (>150 min/week), psychosocial stress, socioeconomic status (below undergraduate level and low income), dietary habits (irregular eating and tea consumption), and opioid use. Biochemical analysis of fasting blood glucose (FBG), HDL-C, and triglyceride (TG) levels was performed using clinical laboratory assay standards. Furthermore, records of hypertension, diabetes mellitus, and surgical procedures were collected.

### Upper Gastrointestinal Endoscopy

Following international standards, an upper endoscopy was performed using a GIF-H260 Gastroscope (Olympus Corp., Tokyo, Japan) before monitoring using 24 hours MII-pH and HRM. A hiatal hernia was identified through upper endoscopy.^9^ Barrett’s esophagus was diagnosed if salmon-colored mucosa was observed during endoscopy, and goblet cells and intestinal metaplasia were detected by histology.^10^

### pH Monitoring

Proton-pump inhibitors and other drugs that affect gastrointestinal function were stopped at least 1 week before the test, and the 24-hour MII-pH test was conducted using an ambulatory MII-pH monitor (Diversatek Healthcare, CO, USA). The calibrated catheter (ZAI-BS-01; Diversatek Healthcare) was positioned in the nasal cavity such that the pH electrode was 5 cm higher than the lower esophageal sphincter (LES) with the 6 impedance channels at 3, 5, 7, 9, 15, and 17 cm positioned proximal to the LES. Patients continued their normal activities while posture, meals, and symptoms were documented. Two researchers used BioView software (Diversatek Healthcare) to analyze the data manually. The following information was gathered: mean nocturnal baseline impedance (MNBI); acid exposure time (AET), total, upright, and prone; total number of reflux episodes (TR); symptom association probability (SAP); symptom index (SI) (positive if ≥50% and positive if ≥95%); and post-reflux swallow-induced peristaltic wave (PSPW) index.

An antegrade frequency decrease of 50% beginning in the proximal channel with movement to the distal channel, returning to a 50% baseline within 30 seconds of reflux, was considered a PSPW.^11^ The TR divided by the PSPW number is the PSPW index.^12^ The MNBI was examined in the most distal channel at night. The MNBI was established by taking the mean of 3 10-minute intervals (about 1, 2, and 3 am); pH-dropping, refluxing, and swallowing times were not included. Following previous studies, the PSPW index, MNBI, and TR thresholds were 50%, 2000, and 40, respectively.^13^^,^^14^

### Esophageal High-Resolution Manometry

An HRM device recorded manometric parameters (Medtronic, MN, USA). A solid-state manometric catheter (4 mm outside diameter, 36 sensors on circumference 1 cm apart) was calibrated from 0 to 300 mmHg and attached to the device. The patient was in a prone position for the measurements. Two trained physicians utilized ManoView software (Medtronic) to conduct a manual and independent data analysis. The results were evaluated through the Chicago Classification (v.4.0).^15^

Ineffective swallows, median integrated relaxation pressure (IRP), mean distal contractile integral (DCI), LES pressure (LESP), and esophageal peristalsis type were among the obtained metrics. Below 39.3 mm Hg/cm, the EGJ contractile integral (EGJ-CI) was considered abnormally low.^15^

The patient took 10 swallows (5 mL) at intervals of 30 seconds before the measurement of primary peristalsis. The strength of esophageal body contractions was assessed using the DCI. Five multiple rapid swallows (MRSs) sequences were performed by steadily injecting water into the mouth using a syringe to further evaluate esophageal function. A minimum of 4 rapid swallows of 2 mL of water were required for each MRS sequence, with a gap of no more than 4 seconds between swallows. The post-MRS DCI: standard single swallow (SS) DCI ratio >1 was considered to indicate the presence of an intact contraction reserve.^16^

Swallowing failures with a DCI <100 mm Hg/cm/s, swallowing failures with a DCI <450 mm Hg/cm/s, weak swallows with a DCI of 100-450 mm Hg/cm/s, and fragmented swallows with peristaltic transition zone defects exceeding 5 cm were all considered ineffective. Based on the Chicago Classification version 4.0, at least 70% of swallows must be ineffective, or 50% must fail to be diagnosed with ineffective esophageal motility (IEM).^15^

### Diagnostic Criteria

Based on the pH of the refluxing liquid, episodes were defined as non-acid (pH >4), weakly acidic (pH 4-7), or acid reflux (pH <4). Reflux that reaches the proximal impedance channel 15 cm above the LES is referred to as proximal reflux. The collected data were evaluated according to Lyon Consensus criteria.^13^ Pathological cases were characterized by a total AET exceeding 6%.

### Definitions

Weight (kg) divided by height^2^ (m^2^) was used to determine the BMI. Waist circumference (cm) divided by hip circumference (cm) represented the waist–hip ratio (WHR). Obesity in East Asians is defined by the WHO as demonstrating a BMI of 25 kg/m^2^ or more.^17^ The Adult Treatment Panel III criteria were utilized to assess metabolic state.^18^ More than 2 of the following characteristics were present in MHO patients: (1) TG ≥ 1.7 mmol/L, (2) FBG ≥ 5.6 mmol/L, (3) HDL-C < 1.29 mmol/L for females or <1.03 mmol/L for males, and (4) diastolic blood pressure ≥85 mm Hg or systolic blood pressure ≥130 mm Hg. Four phenotypes were identified among the participants: BMI ≥ 25 kg/m^2^ and 2 or more metabolic syndrome components are identified as MUO; BMI ≥ 25 kg/m^2^ and 2 or more metabolic syndrome components are identified as MUNO; BMI < 25 kg/m^2^ and 2 or more metabolic syndrome components are identified as MHO; and BMI ≥ 25 kg/m^2^ and < 2 metabolic syndrome components are identified as MHNO.

### Statistical Analysis

For all statistical analyses, SPSS software version 26.0 (IBM SPSS Corp.; Armonk, NY, USA) was used. Data distributions were assessed with Shapiro–Wilk tests, and the 2 groups were compared using the dependent sample *t*-test. The mean ± standard deviation (SD) represents how the data are shown. Categorical variables are given as frequencies and percentages, and chi-squared and Fisher’s exact methods were used to compare the 2 groups. When more than 2 groups were being compared, a 1-way analysis of variance was employed. Waist–hip ratio, HRM parameters, and 24-hour MII-pH parameters were compared using Pearson’s correlation coefficient (2-tailed *P*-value). The associations between various phenotypes, the prevalence of IEM, and pathologic AET were examined using logistic regression. The MUO, MUNO, and MHO groups’ 95% CIs and ORs were determined with the MHNO group as a reference. Three models were utilized; no adjustments were made for Model 1, while Model 2 was adjusted for age, sex, and BMI, and Model 3 for smoking, drinking, waist–hip ratio, psychosocial stress, dietary habits (irregular eating and drinking tea), socioeconomic status (below undergraduate and low income), opioid use, sex, age, and BMI. Two-tailed *P*-values <−.05 were considered statistically significant.

## Results

### Participant Baseline Features

Among the 605 participants, MUO, MUNO, MHO, and MHNO phenotypes were present in 141 (23.3%), 128 (21.2%), 148 (24.4%), and 188 (31.1%) of them, respectively (Table 1). Relative to participants with MHO, MUNO, and MUO phenotypes, those with the MHNO phenotype were generally younger (*P* < .001). No differences in sex were seen among the 4 phenotypes. Individuals with MHO, MHNO, and MUO phenotypes exhibited significantly greater levels of smoking, alcohol consumption, BMI, WHR, psychosocial stress, and socioeconomic status (below undergraduate and low income), as well as irregular dietary habits and tea consumption compared to the MHNO phenotype (*P* < .05). Endoscopy indicated that participants with MHO, MUNO, or MUO phenotypes exhibited RE or a hiatal hernia compared to the MHNO phenotype (*P* < .05). Barrett’s esophagus was observed more frequently in individuals with MHO (8.1%), MUNO (7.8%), and MUO (9.9%) phenotypes compared to those with the MHNO (6.4%) phenotype, although the differences were non-significant (*P* = .706).

### Results of 24-Hour Multichannel Intraluminal Impedance-pH Monitoring

Table 2 presents the pH impedance parameters according to phenotype. Participants demonstrating MUO and MHO phenotypes displayed significantly higher total, upright, and supine AETs, TRs, acid reflux, proximal reflux, and DeMeester scores, alongside a reduced MNBI and PSPW index than the MUNO and MHNO phenotypes. However, other parameters exhibited no substantial variations among the four groups (all *P* < .05).

### High-Resolution Manometry

High-resolution manometry findings are presented according to phenotype in Table 3. In comparison to MHNO and MUNO, MUO and MHO were associated with a reduced LESP and ECJ-CI (*P* < .05), a smaller proportion of ineffective swallows, a diminished occurrence of IEM, and a considerably elevated median IRP. Furthermore, other parameters exhibited insignificant variances among the 4 groups.

### Additional Data from 24-Hour Multichannel Intraluminal Impedance-pH Monitoring and High-Resolution Manometry

**Figure 2.**
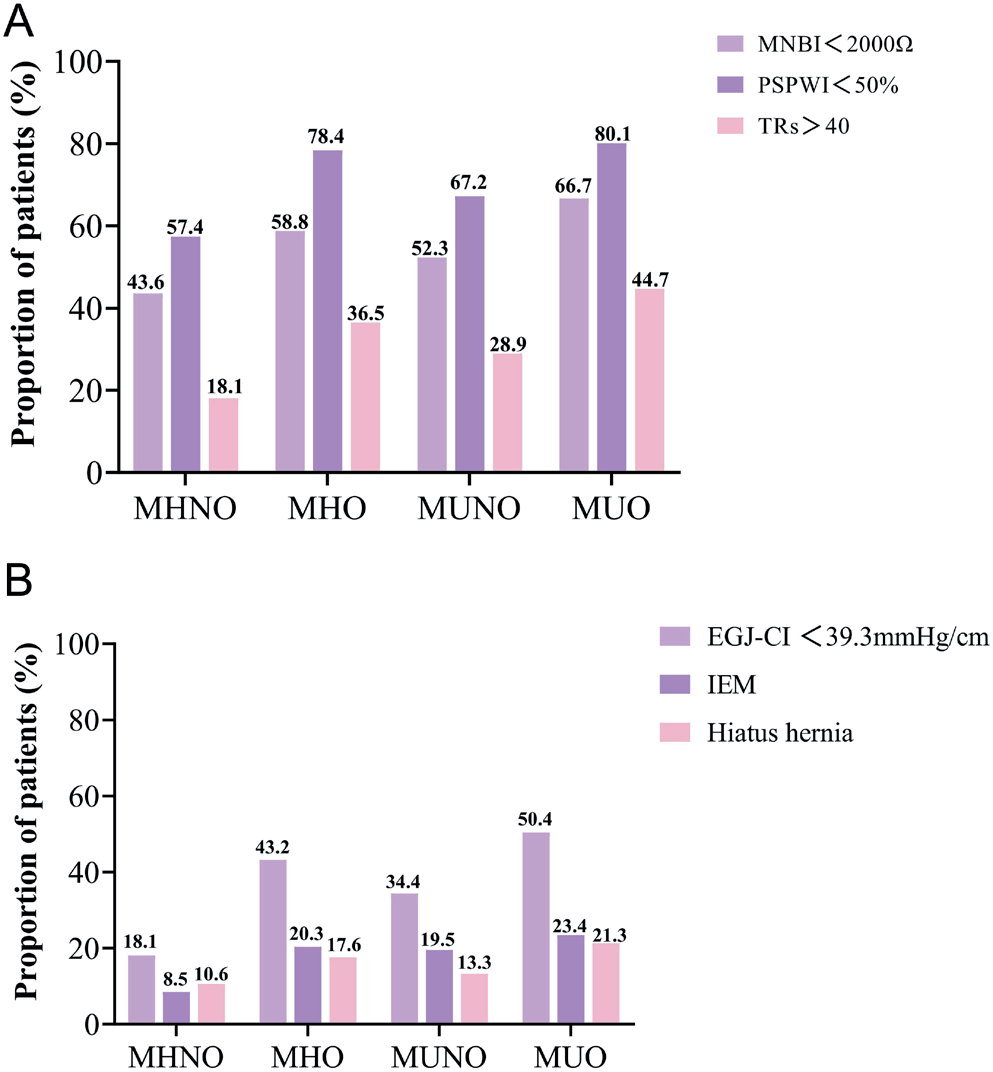
Supplementary data from 24-hour MII-pH monitoring (A) and esophageal high-resolution manometry (B) outcomes for each metabolic obesity phenotype.

Individuals with MUO and MHO phenotypes had significantly lower MNBI and PSPW indexes and higher TRs than patients with MHNO and MUNO phenotypes, according to the adjunctive 24-hour MII-pH data (*P* < .001; Figure 2A). The adjunctive HRM data (Figure 2B) indicated that MHO and MUO participants had higher levels of IEM and hiatal hernia, along with significantly lower ECJ-CI compared to patients with MHNO and MUNO phenotypes (*P* < .05).

### Waist–Hip Ratio, 24-Hour Multichannel Intraluminal Impedance-pH, and High-Resolution Manometry Correlation

**Figure 3.**
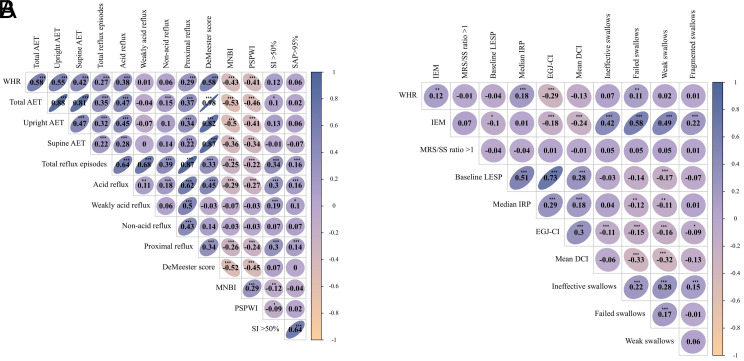
Correlation between the WHR and 24-hour MII-pH monitoring (A) and esophageal high-resolution manometry (B) parameters. *P < .05; **P < .01; ***P < .01.

Correlations between the 24-hour MII-pH and WHR are presented in Figure 3A. DeMeester score (*r* = 0.58, *P* < .001), proximal reflux (*r* = 0.29, *P* < .001), acid reflux (*r* = 0.38, *P* < .001), TRs (*r* = 0.27, *P* < .001), supine AET (*r* = 0.42, *P* < .001), upright AET (*r* = 0.55, *P* < .001), and total AET (*r* = 0.58, *P* < .001) were all substantially favorably linked with the WHR. The MNBI (*r* = −0.43, *P* < .001) and PSPW index (*r* = −0.41, *P* < .001) displayed a strong negative relationship with the WHR.

Figure 3B presents the findings from the correlation analysis between the WHR and HRM. The WHR exhibited a substantial favorable relationship with IEM (*r* = 0.12, *P* = .003), median IRP (*r* = 0.18, *P* < .001), EGJ-CI (*r* = 0.29, *P* < .001), and swallowing failure (*r* = 0.11, *P* = .007).

### Relationship Between Metabolic Obese Phenotype and Risk of Acid Exposure Time

**Figure 4.**
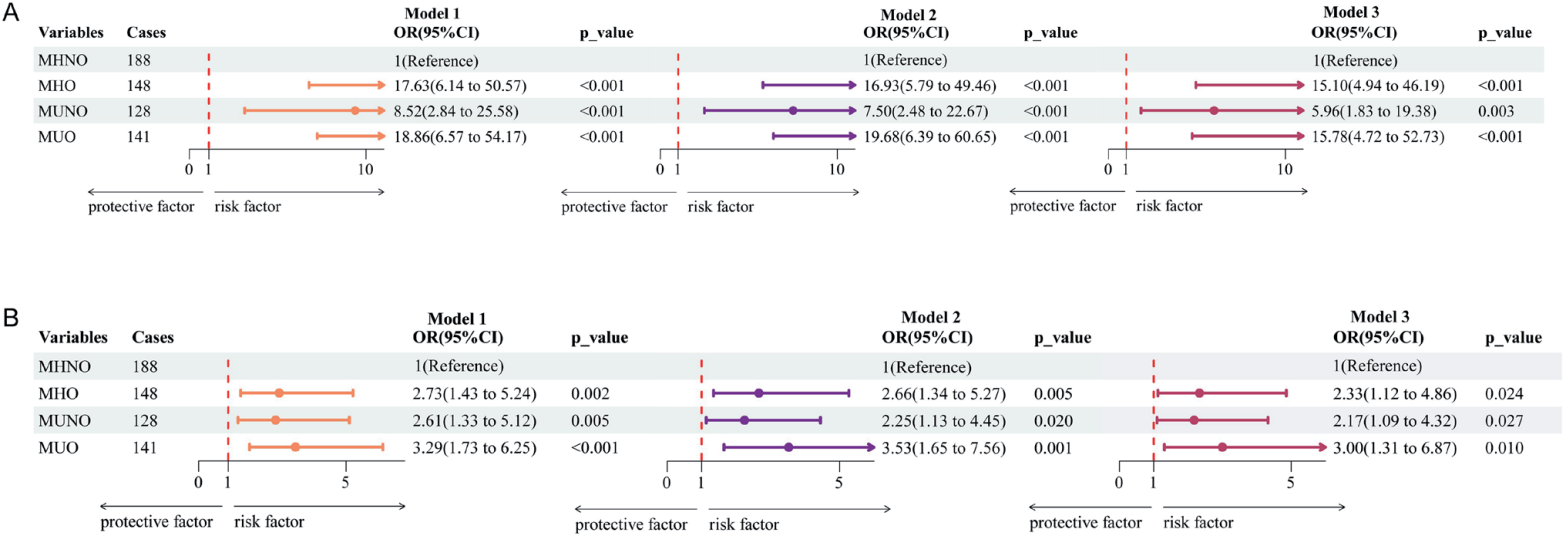
Correlation between metabolic obesity phenotype and the risk of a pathological AET of >6% (A) and ineffective esophageal motility (B). Model 1: not adjusted; Model 2: adjusted for sex, age, and body mass index; Model 3: adjusted for sex, age, body mass index, smoking, alcohol consumption, waist–hip ratio, psychosocial stress, socioeconomic status (below undergraduate and low income), dietary habits (irregular eating and tea consumption), and opioid use.

Metabolically unhealthy obesity, MUNO, and MHO phenotypes were correlated with an increased possibility of having a pathological AET, according to a logistic regression study of AET (Figure 4A; *P* < .05). In Model 3, the MUO, MUNO, and MHO phenotypes had adjusted ORs (95% CI) of 15.78 (4.72-52.73), 5.96 (1.83-19.38), and 15.10 (4.94-46.19) for the prevalence of a pathological AET in comparison to the MHNO phenotype, respectively.

### Association Between Ineffective Esophageal Motility Risk and the Metabolic Obese Phenotype

Metabolically healthy obesity, MUNO, and MUO phenotypes have been attributed to an increased IEM risk, according to logistic regression analysis of IEM (Figure 4B; *P* < .05). The adjusted ORs (95% CI) for IEM for the MHO, MUNO, and MUO phenotypes in Model 3 were, respectively, 3.00 (1.31-6.87), 2.17 (1.09-4.32), and 2.33 (1.12-4.86) relative to the MHNO phenotype.

## Discussion

Relative to the MHNO phenotype, MHO, MUNO, and MUO phenotypes were substantially linked with a greater risk of esophageal reflux and motility dysmotility in the current investigation. Regardless of metabolic health, these hazards were similarly higher in obese individuals relative to those who were not obese. These results offer new insights for managing individuals with various metabolic obesity characteristics.

The abnormalities in esophageal body contractility, LES relaxation issues, and changes in baseline LESP are manometric anomalies in obese patients that might be linked to obesity-induced elevated intra-abdominal pressure.^19^ Reduced LESP experienced by obese people compromises the anti-reflux barrier and causes gastroesophageal reflux to occur.^19^ The current research found that MUO and MHO phenotypes were associated with a higher RE and BE prevalence than MHNO and MUNO phenotypes. Individuals with MHO and MUO phenotypes exhibited a greater likelihood of experiencing pathological AET and IEM, potentially contributing to the increased prevalence of esophageal symptoms in obese populations.

There is a deficiency in research employing objective tests to evaluate esophageal reflux and motility function in individuals with metabolic disorders. The 24-hour pH-impedance test revealed marked increases in all reflux indicators in participants with MUO and MHO phenotypes compared to participants with MUNO and MHNO phenotypes, indicating a probable association between obesity and pathological reflux. Additional assessment of pertinent objective parameters is essential to establish a standardized diagnostic instrument for GERD in participants with diverse metabolic obesity phenotypes. Not much research has been done on the diagnostic efficacy of TR in people with the MHO phenotype and usual reflux symptoms. The TR physiological threshold represents the new standard.^13^ Our investigation revealed that TR positivity was more prevalent in individuals with MUO and MHO phenotypes than in those with MUNO and MHNO phenotypes, suggesting that obesity may exacerbate reflux.

The PSPW index indicates esophageal chemical clearance, representing a defense against reflux induced by the esophagus-salivary reflex. Compared to the traditional TR, AET, and MII-pH parameters, the PSPW index demonstrates superior sensitivity and overall accuracy in diagnosing GERD.^12^ Another study^20^ indicated that obese patients exhibited diminished esophageal sensitivity to acid perfusion, which may influence esophageal clearance. The results indicated a markedly reduced PSPW index in participants exhibiting MUO or MHO phenotypes compared to those with MUNO or MHNO. Obese patients typically exhibit diminished esophageal clearance relative to non-obese patients, potentially elucidating their increased vulnerability to GERD.

A sign of pathological reflux, standard impedance suggests the health of the esophagus mucosa. In this study, patients exhibiting the MHO, MUO, and MUNO phenotypes demonstrated lower MNBI compared to those with the MHNO phenotype. Blevins et al^21^ explored the association between obesity and abnormal esophageal MNBI and hypothesized that obesity alters esophageal barrier function. Our study found that over two-thirds of individuals with the MHO phenotype exhibited an abnormal MNBI, indicating significant esophageal mucosal impairment in these patients. The exact mechanism contributing to this impairment in obesity is not yet understood. Similar research^22^ has demonstrated that obesity increases fluorescein leakage, decreases desmosomal density, and increases intercellular space, compromising the esophageal barrier’s functional and structural stability. Furthermore, the current investigation discovered a favorable relationship between DeMeester scores and metabolic obesity phenotypes. Schneider et al^23^ reported that DeMeester scores differed significantly among various stages of obesity. These findings indicate an association between obesity and abnormal acid reflux. A separate study revealed a reduced rate of non-acid reflux in non-obese patients (BMI < 25 and 25-30 kg/m^2^) relative to obese patients (BMI > 30 kg/m^2^).^24^ In our investigation, patients with various metabolic obesity phenotypes had equal frequencies of non-acid and weakly acid reflux; nevertheless, there was a strong association between phenotype and acid reflux. Ultimately, our analyses support the early administration of antacid therapy to reduce acid reflux in obese patients with GERD.

As determined by HRM, esophageal function varies among metabolically obese phenotypes. Braghetto et al^25^ reported lower LESP values in obese patients, which were associated with increased pathogenic gastroesophageal reflux and reflux esophagitis. Similarly, the present results showed that baseline LESP was substantially lower in individuals with MUO and MHO phenotypes than in those with MUNO and MHNO phenotypes. This phenomenon may result from heightened temporary LES relaxation in individuals exhibiting the MHO phenotype. Acid exposure and total reflux events have been adversely correlated with the new EGJ-CI measure, which measures barrier function.^26^ The current study found that MUO phenotype patients had lower EGJ-CI values than MHNO phenotype patients, and MHO and MUO patients showed a greater probability of hypotensive ECJ-CI. These findings corroborate earlier research indicating that obesity significantly impacts esophageal function. Specifically, it changes EGJ morphology, leading to a higher acid reflux burden. Furthermore, people with hiatal hernias are most likely to have the MUO phenotype.^13^

In order to diagnose esophageal motility problems, the median IRP is required. In the present study, participants with MUO and MHO phenotypes had a higher median IRP than people with MHNO and MUNO phenotypes. Obese patients had significantly elevated LES IRP-4s compared to healthy subjects, as per the Yen et al. study^27^ In obese patients, an elevated DCI should offset the increased LES IRP-4s, leading to a higher incidence of esophageal dysmotility disorders. However, the mean DCI in our study lacks significant variations, likely attributable to the limited number of participants classified as morbidly obese (BMI ≥ 35 kg/m^2^). After controlling for all variables, the IEM prevalence was markedly greater in the MUNO or MHO phenotypes than the MHNO phenotype, showing that obesity is a major risk factor for IEM, irrespective of metabolic phenotype. Research demonstrates that hypercontractility prolongs esophageal acid exposure, therefore elevating esophageal mucosal damage risks.^28^ Ineffective esophageal motility heightens the risk of increased AET by diminishing contractile strength and augmenting acid load.

The present research has various limitations. Initially, the single-center and retrospective design of the study constrained its power and generalizability, introduced potential confounding variables, and precluded the establishment of causality. Furthermore, the role of humoral factors, including insulin, leptin, and adipokines, in the contribution of visceral adipose tissue to the development of GERD was not investigated. Heartburn and regurgitation, the principal symptoms of GERD, were examined. These findings necessitate validation in patients exhibiting extraesophageal reflux symptoms.

In summary, individuals exhibiting the MHO phenotype demonstrated an elevated IEM and GERD risk compared to those with the MUNO phenotype. This indicates that weight management should be advised for all obese individuals, irrespective of their metabolic classification.

## Figures and Tables

**Table 1.** Patient Features Based on the Metabolic Obese Phenotype

Variable	MHNO	MHO	MUNO	MUO	*P*
Number of participants	188 (31.1)	148 (24.4)	128 (21.2)	141 (23.3)	
Age (years)	50.33 ± 14.69	53.01 ± 14.81	56.17 ± 13.75	59.67 ± 14.85	<.001
Male sex, n (%)	64 (34.0)	68 (45.9)	51 (39.8)	64 (40.4)	.092
Smoking, n (%)	26 (13.8)	31 (20.9)	40 (31.3)	57 (40.4)	<.001
Alcohol consumption, n (%)	27 (14.4)	36 (24.3)	41 (32.0)	48 (34.0)	<.001
Physical activity, n (%)	92 (48.9)	53 (35.8)	42 (32.8)	40 (28.4)	.001
BMI (kg/m^2^)	21.64 ± 2.71	26.12 ± 6.41	21.93 ± 2.30	30.01 ± 7.80	<.001
WHR	0.84 ± 0.011	0.86 ± 0.015	0.84 ± 0.014	0.86 ± 0.015	<.001
Psychosocial stress, n (%)	32 (17.0)	39 (26.4)	45 (35.2)	56 (39.7)	<.001
Socioeconomic status					
Below undergraduate, n (%)	44 (23.4)	45 (30.4)	52 (40.6)	65 (46.1)	<.001
Low income, n (%)	30 (16.0)	36 (24.3)	46 (35.9)	56 (39.7)	<.001
Dietary habits					
Irregular eating, n (%)	16 (8.5)	26 (17.6)	35 (27.3)	48 (34.0)	<.001
Tea consumption, n (%)	18 (9.6)	42 (28.4)	50 (39.1)	57 (40.4)	<.001
Use of opioids, n (%)	9 (4.8)	20 (13.5)	20 (15.6)	30 (21.3)	<.001
Endoscopy n (%)					
Reflux esophagitis	20 (10.6)	28 (18.9)	22 (17.2)	40 (28.4)	.001
Barrett’s esophagus	12 (6.4)	12 (8.1)	10 (7.8)	14 (9.9)	.706
Hiatal hernia	20 (10.6)	26 (17.6)	17 (13.3)	30 (21.3)	.046

MHNO, metabolically healthy non-obesity; MHO, metabolically healthy obesity; MUNO, metabolically unhealthy non-obesity; MUO, metabolically unhealthy obesity; WHR, waist hip ratio.

**Table 2.** The 24-hour MII-pH Data Classified by Each Metabolic Obese Phenotype

Variable	MHNO	MHO	MUNO	MUO	*P*
Number of participants	188 (31.1)	148 (24.4)	128 (21.2)	141 (23.3)	
AET (%)					
Total AET (%)	1.24 ± 2.03	4.49 ± 4.79	2.92 ± 4.63	5.50 ± 6.21	<.001
Upright AET (%)	1.86 ± 3.77	6.22 ± 6.89	3.72 ± 5.29	7.00 ± 7.74	<.001
Supine AET (%)	0.64 ± 1.74	2.95 ± 5.91	1.93 ± 5.29	4.07 ± 7.10	<.001
Reflux episodes (n)					
Total reflux episodes (n)	23.87 ± 21.21	38.32 ± 27.66	29.91 ± 22.40	42.72 ± 39.63	<.001
Acid reflux (n)	12.17 ± 12.81	25.32 ± 28.56	17.83 ± 18.05	25.48 ± 19.86	<.001
Weakly acid reflux (n)	11.77 ± 16.11	13.14 ± 17.45	11.38 ± 11.16	15.25 ± 27.57	.300
Non-acid reflux (n)	1.04 ± 2.40	0.90 ± 1.96	0.70 ± 2.03	1.99 ± 12.32	.319
Proximal reflux (n)	8.79 ± 10.69	17.03 ± 15.96	12.69 ± 15.10	21.00 ± 23.60	<.001
DeMeester score	5.11 ± 7.46	16.55 ± 17.00	11.03 ± 16.38	19.77 ± 21.61	<.001
MNBI (Ω)	2173.43 ± 742.67	1833.74 ± 749.60	1924.38 ± 715.71	1602.84 ± 752.80	<.001
PSPWI (%)	46.41 ± 16.58	37.35 ± 17.03	41.83 ± 20.58	30.56 ± 18.18	<.001
SI>50%	103 (54.8)	93 (62.8)	76 (59.4)	87 (61.7)	.442
SAP>95%	120 (63.8)	101 (68.2)	82 (64.1)	84 (59.6)	.502

MHNO, metabolically healthy non-obesity; MHO, metabolically healthy obesity; MUNO, metabolically unhealthy non-obesity; MUO, metabolically unhealthy obesity; AET, acid exposure time; MNBI, mean nocturnal baseline impedance; PSPWI, post-reflux swallow-induced peristaltic wave index; SI, symptom index; SAP, symptom association probability.

**Table 3.** Outcomes of Esophageal HRM Categorized by Each Metabolic Obesity Phenotype

Variable	MHNO	MHO	MUNO	MUO	*P*
Number of participants	188 (31.1)	148 (24.4)	128 (21.2)	141 (23.3)	
MRS/SS ratio >1, n (%)	81 (43.1)	68 (45.9)	60 (46.9)	58 (41.1)	.756
Baseline LESP (mm Hg)	14.65 ± 7.31	15.31 ± 8.50	13.29 ± 7.39	17.59 ± 8.84	<.001
Median IRP (mm Hg)	5.16 ± 4.18	10.66 ± 4.85	7.66 ± 4.13	11.65 ± 5.16	<.001
EGJ-CI (mm Hg/cm)	63.11 ± 24.54	47.46 ± 26.67	53.47 ± 26.84	44.80 ± 29.73	<.001
Mean DCI (mm Hg/cm/s)	1958.27 ± 1581.88	1758.12 ± 1385.42	1747.63 ± 2045.65	1933.73 ± 1637.33	.556
Ineffective swallows (%)	22.50 ± 30.16	31.69 ± 36.78	29.06 ± 32.95	33.12 ± 37.65	.023
Failed swallows (%)	10.00 ± 19.43	12.57 ± 23.24	11.64 ± 18.56	8.16 ± 17.39	.250
Weak swallows (%)	9.04 ± 18.30	7.64 ± 16.39	10.00 ± 17.25	9.43 ± 17.60	.703
Fragmented swallows (%)	2.45 ± 8.49	2.09 ± 7.67	2.11 ± 7.17	3.05 ± 9.85	.754
IEM, n (%)	16 (8.5)	30 (20.3)	25 (19.5)	28 (21.2)	.002

MHNO, metabolically healthy non-obesity; MHO, metabolically healthy obesity; MUNO, metabolically unhealthy non-obesity; MUO, metabolically unhealthy obesity; IEM: ineffective esophageal motility.

## Data Availability

The data that support the findings of this study are available on request from the corresponding author.
